# Sex differences in taste neophobia and conditioned aversion across fluid administration methods

**DOI:** 10.3389/fnbeh.2026.1761664

**Published:** 2026-03-10

**Authors:** Ron Gerbi, Oran Rahamim, Elor Arieli, Amit Worcel, Anan Moran

**Affiliations:** 1 Department of Neurobiology, School of Neurobiology, Biochemistry & Biophysics, The George S. Wise Faculty of Life Science, Tel-Aviv University, Tel Aviv, Israel; 2 Sagol School of Neuroscience, Tel Aviv University, Tel Aviv, Israel

**Keywords:** conditioned taste aversion, decision making, food administration, learning, neophobia, rat, sex differences, taste

## Abstract

Rodent studies of the taste system commonly employ two methods of taste administration (MOA): active licking from spouts or intra-oral cannula (IOC) deliveries. While bottle drinking preserves natural consumption behavior, IOC administration, where animals receive liquids passively into their oral cavity, provides precise temporal control of stimulus delivery but limits the reliability of measuring voluntary intake and hedonic response. To overcome these limitations, a third method, nose-poke for IOC delivery (NP-IOC), was introduced. In NP-IOC, each taste is delivered through the IOC following an active nose poke, thus combining voluntary decision-making with temporal precision. Whether NP-IOC preserves natural taste-guided behavior, however, remains unknown. Here, we examined how NP-IOC affects taste neophobia (the reluctance to consume novel tastes) and conditioned taste aversion (CTA, avoidance of a taste paired with malaise). Rats received water either *via* standard bottle licking (control) or through the NP-IOC system. Following habituation, animals were tested for neophobia using low-neophobic (LN) sucrose or high-neophobic (HN) saccharin solutions, followed by CTA training *via* lithium chloride injection. Our results showed sexual differences in neophobia using NP-IOC: males preserved the expected difference between LN and HN tastes, whereas females showed attenuated neophobia, eliminating the typical HN avoidance observed with bottle administration. Nevertheless, CTA learning remained robust across sexes and MOAs. Deeper analysis of this seemingly similar learned aversion, however, revealed again sex differences: while male rats showed strong CTA regardless of pre-CTA consumption, females maintained a correlation between pre and post-CTA consumption under both MOAs, suggesting sex-specific taste learning patterns. These findings support the use of NP-IOC in taste research that requires both precise stimulus control and voluntary behavior, while also underscoring the necessity of exploring divergent behavioral strategies and the associated brain circuits in males and females.

## 1 Introduction

The rodent taste system has been used for decades to study the mechanisms underlying decision-making and learning. One of its key advantages is its evolutionary relevance, as experimental paradigms in the lab closely mirror the feeding-related decisions rodents make in the wild. When encountering potential food, omnivores like rodents and humans integrate innate and experienced knowledge to optimize their response, ranging from avid consumption when food is deemed palatable and nutritious, to complete rejection if perceived as unpalatable or life-threatening (Rozin and Kalat, 1971). To study decision making and its neural brain mechanisms, laboratory experiments replicate these natural feeding decisions, offering rodents different food options and measuring their response in controlled conditions. These experiments have revealed rodent behaviors strikingly similar to those in humans. For example, when presented with a novel food, rodents often hesitate to consume it, exhibiting “Taste neophobia” (Barnett, 1958; Corey, 1978; Reilly, 2018), a protective mechanism against potential toxicity (Barnett, 1958; Reilly, 2018). Additionally, the outcome of the novel taste consumption dictates further decisions: if the taste is followed by gastric distress, rodents learn to associate the malaise with the specific taste and subsequently avoid consuming it in the future, a phenomenon known as conditioned taste aversion (CTA) (Garcia et al., 1955). Both taste neophobia and CTA serve as fundamental models for studying food-related decision-making, and have been extensively used to uncover the molecular pathways and neuronal circuits that regulate these behaviors (Berman and Dudai, 2001; Bureš et al., 1998).

Depending on the objective of the experiment, the design may favor the use of either solid or liquid stimuli, with liquids generally preferred due to their ease of quantification and delivery. Various methods of administration (MOA) of liquid stimuli have been developed based on the focus of the study. For experiments investigating behavioral responses, researchers typically analyze total consumption or the timing of licks. In contrast, studies targeting specific brain regions or circuits (e.g., in optogenetics or electrophysiology) require precise external control over the timing of taste delivery. The most common method for investigating behavioral responses involves using a spout-equipped bottle or a pipette to administer liquids within a defined time frame (Nachman and Ashe, 1973; Rosenblum et al., 1993). Adding an infrared beam in front of the spout (Silva et al., 2024), with or without mechanisms to control access (like in the Davis rig) (Smith, 2001), enables the collection of precise lick timing, which can be analyzed to account for neophobia (Monk et al., 2014). These MOAs, however, primarily provide information on the total amount consumed and the timing of consumption, but do not allow for fine control of more complex fluid delivery parameters, such as inter-taste stimulus time intervals, different contingencies of fluids from the same spout, or imposing dry licks.

Fine temporal control of fluid delivery is necessary in studies that employ electrophysiological recording to investigate neuronal activity underlying taste perception and memory. These studies often use passive taste administration via an intraoral cannula (IOC)—a thin tube implanted under the skin in the cheek that reaches the animal’s oral cavity (Moran and Katz, 2014; Phillips and Norgren, 1970; Yamamoto et al., 1989). In this MOA, equal-sized portions of taste solutions are passively delivered at specific times, forcing the animal to either swallow or reject the taste. Since this method prevents measurement of consumption-based behavior, researchers commonly use the taste reactivity test (TRT), which involves analyzing stereotyped body and facial responses associated with acceptance or rejection, captured from recorded videos (Grill and Norgren, 1978). The TRT, however, has several limitations; tracking the facial expressions of freely moving animals is technically challenging, the analysis can be time-consuming, and distinguishing between disgust responses and fear of toxicity can be difficult (Dwyer, 2012). Moreover, the forced IOC method bypasses the animal’s natural decision to approach or avoid the solution, leaving only the final decision—whether to consume or reject the taste. To overcome these limitations, a self-administration MOA was introduced, allowing animals to actively initiate taste deliveries (e.g., by nose poking), while the taste is delivered through the IOC in a timed-control manner (Arieli et al., 2020; Yamamoto et al., 2002). Active nose-poking for IOC (NP-IOC) fluid delivery MOA successfully satisfies both requirements: precise quantification of behavior (by counting the number of pokes or dry licks) and accurate control over taste stimulation timing (regulated by the delivery system). Another advantage of the NP-IOC technique is the controlled inter-trial interval: while natural free licking has an inter-lick interval of approximately 150 ms (Davis, 1996), the NP-IOC method extends this interval to the order of seconds if desired, enabling a more precise analysis of individual taste trials, independent of preceding ones.

Despite its advantages, the NP-IOC technique may introduce biases compared to more natural MOAs. Specifically, its unnatural nature may engage different cognitive functions through different brain circuits, which together might show attenuation of the natural taste-related decisions. Such behavioral attenuation has been observed in studies showing differences in CTA expression and faster extinction in rats that were passively infused intraorally with saccharin via the IOC method compared with rats that freely drank from a bottle (Fouquet et al., 2001; Wolgin and Wade, 1990). Understanding the influence of MOAs on taste-related behaviors is particularly important in studies that integrate multiple MOAs within the same experiment. For instance, one study conditioned sucrose using different MOAs but tested CTA using free drinking, resulting in different CTA extinction rates (Yamamoto et al., 2002). At the neural level, distinct circuits appeared to mediate CTA depending on the MOA used. For example, basolateral amygdala lesions were found to eliminate CTA when the taste was passively infused but only mildly attenuate learning when the taste was actively licked from a bottle (Schafe et al., 1998). Together, these findings highlight the critical role of taste MOAs in shaping both the acquisition and extinction of CTA.

In addition to methodological considerations, accumulating evidence points to sex differences in taste learning and related behaviors. Although CTA is highly robust across experimental conditions, studies in other learning domains suggest that males and females may rely on partially distinct neural mechanisms (Dalla and Shors, 2009; Greiner et al., 2024), and differences in innate preferences and sensitivities have also been reported (Curtis et al., 2004; Flynn et al., 1993). However, most work on sex differences in taste learning of rodents has employed a single method of consumption (typically spouted bottles or pipettes) (Angulo and Arévalo-Romero, 2021; Nakajima and Li, 2024), leaving largely unexplored how sex differences may interact with different MOAs. Highlighting such interactions is critical not only for understanding variability in the behavior of the species, but also for interpreting how methodological factors, such as the MOA, might shape sex-specific outcomes in taste learning.

In the current study, we investigated the impact of different MOAs on neophobia and CTA in rats. Specifically, male and female rats consumed either sucrose (low-neophobic stimulus) or saccharin (high-neophobic stimulus) through a traditional spouted bottle or *via* NP-IOC, followed by a CTA protocol to monitor changes in consumption. Our results revealed that neophobic responses were selectively altered in females under NP-IOC, while CTA learning averages remained robust across conditions. Deeper analysis, however, revealed differences in the strategies used by males and females following CTA. Thus, the NP-IOC method not only affords precise behavioral control in taste learning studies but also exposes sex-specific dynamics in neophobia and aversion, underscoring its value for linking neural mechanisms with individual variability in consumptive behavior.

## 2 Methods

### 2.1 Animals

Subjects were female (210-275 g) and male (232–406 g) Long Evans rats, 4–6 months old at the beginning of the experiment, who were all bred and maintained as littermates in groups of 2–4 in a 12-h light/dark cycle in the animal facility at Tel Aviv University. Rats were divided into two experimental groups determined by the two MOAs we investigated: NP-IOC and bottle. Rats were isolated in separate home cages and handled for 5 days before their surgery (NP-IOC group) or experimental sessions (bottle group) to reduce stress during experiments. Rats that underwent surgery were given a week to recover with wet food pellets and ad libitum access to water before the beginning of the experiment. The rats were weighed on day 1 and throughout the habituation and experimental days to monitor proper welfare.

### 2.2 Taste stimuli

Two sweet solutions were used in this experiment: 0.2 M sucrose [low neophobic (LN)], and 0.15% saccharin [higher neophobic (HN)]. Each tasting compound was dissolved and mixed in tap water to produce its chosen concentration on the morning of each experimental day.

### 2.3 Surgical procedures

Animals were briefly anesthetized with Isoflurane (0.5 mL/300 g) followed by an intraperitoneal (IP) injection of a ketamine xylazine mixture (KX, 100 mg/kg ketamine and 10 mg/kg xylazine). Booster injections of KX (one-third of the induction dose) were given as needed throughout the surgery. The head of the anesthetized animal was shaved and secured on a stereotaxic frame to ensure the sturdiness of the skull. After the head was secured, the skull was exposed and thoroughly dried with 30% hydrogen peroxide. Four screws were then inserted into the rat’s skull and covered with dental cement. One IOC (Polyethylene tubing, #802500, AM-systems) was unilaterally inserted lateral to the second molar tooth and underneath the skin to be exposed on the top of the rat’s head. The IOC was combined with a plastic cap, which was sealed with a dust cap to prevent infections and secured on the rat’s head with dental cement. The IOC was inserted on the right or the left side of the rat’s face to avoid placement bias of the IOC. After surgery, the rats were given subcutaneous (S.C.) post-operative injections of saline (0.9% NaCl) for proper hydration, antibiotics (5 mg/kg of Baytril 5%), and pain treatment (1 mg/kg of Meloxicam 0.5%). The rats were given a week of recovery during which a metal rod was inserted into their IOC to prevent blockage of the tube.

### 2.4 Behavioral procedures

#### 2.4.1 Bottle

*Habituation*: In the free-drinking experiment, each morning of the first 3 days, the rats were put in an experimental cage and were allowed to drink for 20 min from a bottle filled with 25 mL of tap water. The weights of the bottles were measured before and after each experimental session throughout all experimental days to calculate the total amount of water the rats consumed. The experimental cages were cleaned thoroughly with 70% ethanol between each experimental session. Four hours later, the rats were given free access to water in their home cages from a standard drinking bottle for an additional 40 min, to allow proper hydration.

*CTA training (Pre-CTA)*: The 4th day of the experiment was similar to the habituation days, except that either 0.2 M sucrose or 0.15% saccharin solution was given instead of water in the bottle. After the drinking session, the rats were injected S.C. with a Lithium Chloride (LiCl) solution (0.3 M in double distilled water, 1% body weight) to induce gastrointestinal malaise. The rats were then returned to their home cages and their consumption was measured, followed by the same delayed free-access bottle drinking session described above.

*CTA test (post-CTA)*: Rats were given 20 min access to 25 mL of the tastant they were previously exposed to on day 4 (LN or HN) in the experimental cage. The rats were then put back into their home cage and the bottles were weighed.

#### 2.4.2 NP-IOC liquid delivery

**Figure 1:**
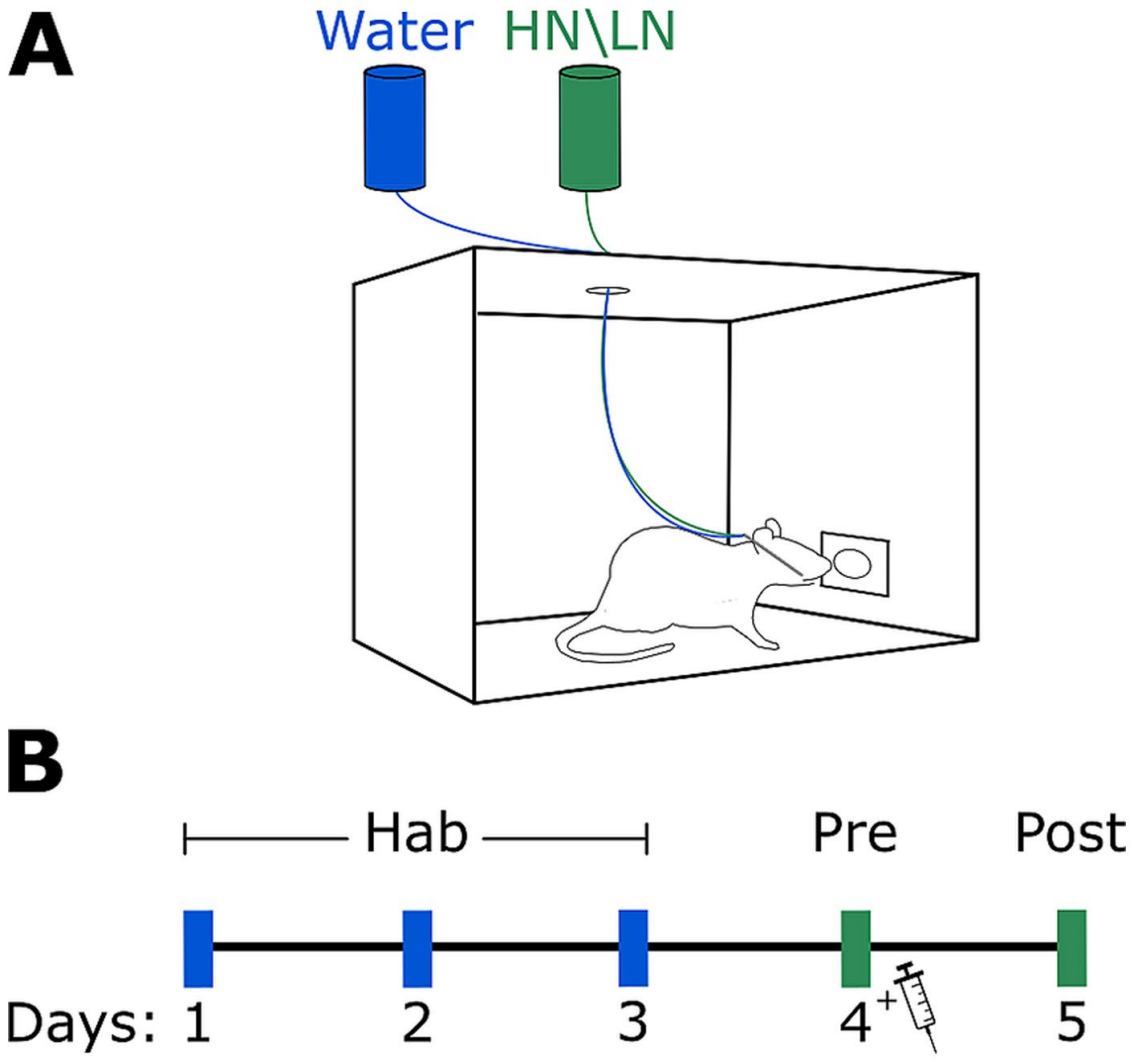
Experimental design. **(A)** Schematic of the cage used for NP-IOC. A nose poke is detected by an infrared beam, which triggers the delivery system to open the appropriate valve and dispense a drop of liquid directly into the rat’s oral cavity. **(B)** Schematic of the CTA procedure. Rats were first habituated to the consumption protocol for three days. On Day 4, they received a novel taste solution, immediately followed by a LiCl injection to induce aversion. On Day 5, the same solution was re-presented to assess aversion strength.

*Habituation:* For this experiment, we used a NP-IOC method for taste administration; during the first 3 days of the experiment the rats were habituated to poke in an infrared-operated nose poke (Coulbourn Instruments, PA, United States) to receive 40 μL drops of water delivered through the IOC directly into their oral cavity. Figure 1A shows the general structure of the experimental setup. The rats were able to receive a water drop with a minimal interval of 3 s, and each water delivery was recorded using a RHD2000 acquisition system (Intan Technologies, CA, United States). The experimental cages were cleaned thoroughly with 70% ethanol between each experimental session. Four hours later, the rats were given access to water in the home cage, following a procedure identical to that used for the bottle-drinking group.

*CTA training (Pre-CTA)*: the 4th day was similar to the habituation days, except that the water was replaced as a stimulant with either LN or HN solution. After 20 min, the rats were taken out of the cage and injected S.C. with 0.3 M LiCl (1% body weight) to induce gastrointestinal malaise and were put back into their home cage, followed four hours later by the same delayed free-access bottle drinking session described above.

*CTA test (Post-CTA)*: The rats underwent the same procedure as that of day 4 with the same tastant (either LN or HN). After the test session, the rat was put back into their home cage and the IOC taste system was properly cleaned with 70% ethanol and water.

### 2.5 Data presentation

#### 2.5.1 Inclusion criterion

Only rats that consumed at least 2 mL of water on the 3rd habituation day (either by licking in the bottle group, or poking at least 50 times in the NP-IOC group) were included in the experimental cohort. Among the 29 rats tested with the nose-poke system, 4 did not meet this inclusion criterion, all of which were females. All rats in the bottle group successfully consumed more than 2 mL water on day 3, and therefore, all were included in the analyses.

#### 2.5.2 Normalized neophobia (NN)

To quantify neophobic behavior, we calculated an NN index representing the reluctance to consume a novel taste relative to water consumption. The index was defined as:


$$\mathrm{NN}=1-(\text{PreCTA}/\text{Wate}r_{\mathrm{Day}3})$$ (1)

Where *PreCTA* represents the amount of novel taste consumed on Day 4, and *Water_Day3_* is the amount of water consumed on the preceding day. Higher NN values indicate stronger neophobic reactions.

#### 2.5.3 Aversion index (AI)

To assess the strength of CTA learning, we calculated an AI, defined as:


AI=1−(PostCTA/PreCTA) (2)

Where *PostCTA* denotes consumption of the conditioned taste on Day 5 and *PreCTA* represents consumption prior to conditioning on Day 4. Higher AI values reflect stronger aversive learning and greater reductions in consumption following conditioning.

### 2.6 Statistical analysis

Statistical analyses were conducted using 3-way ANOVA (Sex × MOA × Taste). Assumptions of residual normality and homogeneity of variance were assessed for all dependent variables prior to analysis using Shapiro–Wilk and Fligner-Killeen tests, respectively; accordingly, parametric factorial ANOVA was conducted. *Post hoc* comparisons were performed using independent t-tests, with correction for multiple comparisons applied using the Hommel procedure where appropriate. An effect was considered statistically significant if *p* < 0.05. To the relationship between Pre and Post-CTA consumption, a Pearson’s correlation test was performed. All statistical analyses were conducted using custom Python scripts, utilizing the Scipy and Statsmodels libraries. Results are presented as mean ± SEM.

## 3 Results

### 3.1 The impact of NP-IOC MOA on water consumption

**Figure 2:**
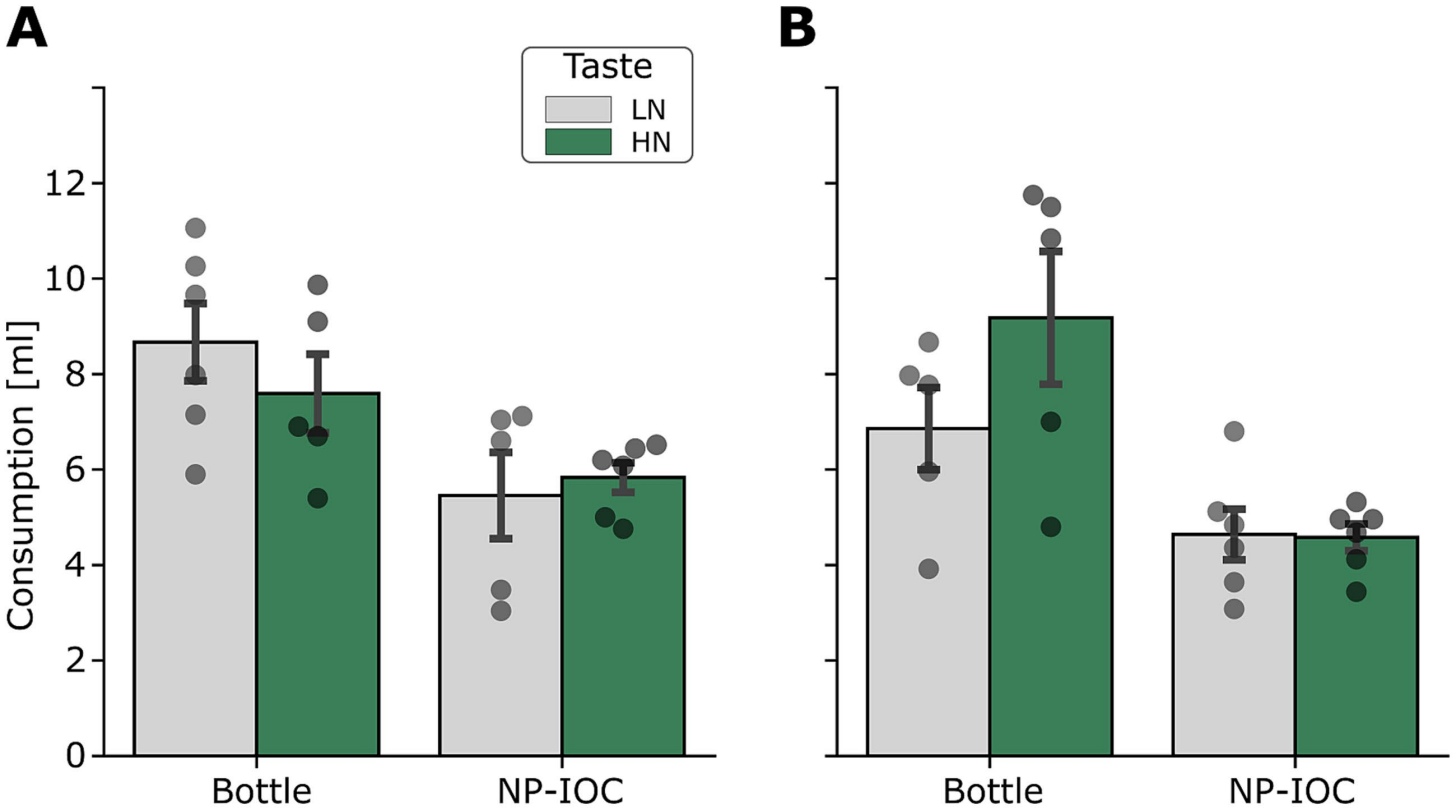
Water consumption at the end of habituation (Day 3) in Bottle and NP-IOC rats, shown separately for males **(A)** and females **(B)**. LN, low-neophobia solution; HN, high-neophobia solution. Data are represented as mean ± SEM.

Rats were randomly assigned to one of the sex-specific groups (rats consuming HN saccharin solution or LN sucrose solution, delivered *via* either MOA), resulting in a total of 8 groups (*females*: bottle *n* = 10, NP-IOC *n* = 12 NP-IOC; *males*: bottle *n* = 11, NP-IOC *n* = 11). To allow direct comparison between consumptions across the two MOAs, we converted the number of pokes to milliliters using the calibrated drop volume (see Methods for details). Before testing for the impact of the MOAs on taste-related behaviors, we wanted to assess whether it has any impact on basal consumption of water. To test that, we compared the water consumption on the 3rd day of the experiment, after the rats had habituated to the drinking regimen. We have found that across the sexes, rats consumed more water from the bottle than from the NP-IOC (Figure 2; three-way ANOVA (Sex × Taste × MOA): *F*_MOA_ (1, 36) = 29.22, *p* < 0.0001), however, there was no difference between water consumption between males and females (Figure 2; three-way ANOVA (Sex × Taste × MOA): *F*_Sex_ (1, 36) = 1.36, *p* = 0.2). Together, these results show that the 20-min consumption window of our experiment, the NP-IOC method led to lower fluid intake compared with the bottle consumption. While this finding highlights the effect of the MOA on baseline consumption, it is important to note that subsequent analyses of neophobia and conditioned aversion relied on normalized measures, therefore accounting for these baseline differences.

### 3.2 The impact of NP-IOC MOA on taste neophobia

Next, we investigated how the MOA modulates taste neophobia in male and female rats, quantified using a NN index (Equation 1). A three-way ANOVA was conducted with Sex (male *vs.* female), Taste (low *vs.* high-neophobic), and MOA (bottle *vs.* NP-IOC) as factors. This analysis revealed a significant three-way interaction between Sex, Taste, and MOA, *F*_Sex × Taste × MOA_ (1, 36) = 4.35, *p* = 0.044, indicating that the effect of MOA on neophobic responses to the two tastes differed between males and females. As expected, the ANOVA also revealed a main effect of Taste [*F*_Taste_ (1, 36) = 26.3, *p* < 0.0001], reflecting the designed differences between low and high neophobic tastes used. A somewhat expected result of the ANOVA was a significant difference found between the MOAs with higher consumption from the bottles than from the NP-IOC [MOA main effect, *F*_MOA_ (1, 36) = 4.25, *p* = 0.046]. This difference is probably the result of the pace of the fluid delivery, which is faster with the active licking. Nevertheless, since all our measures of behavior are relative to the consumption of the same rat, we believe this difference between the MOAs is not critical to our results assessment and conclusions.

**Figure 3:**
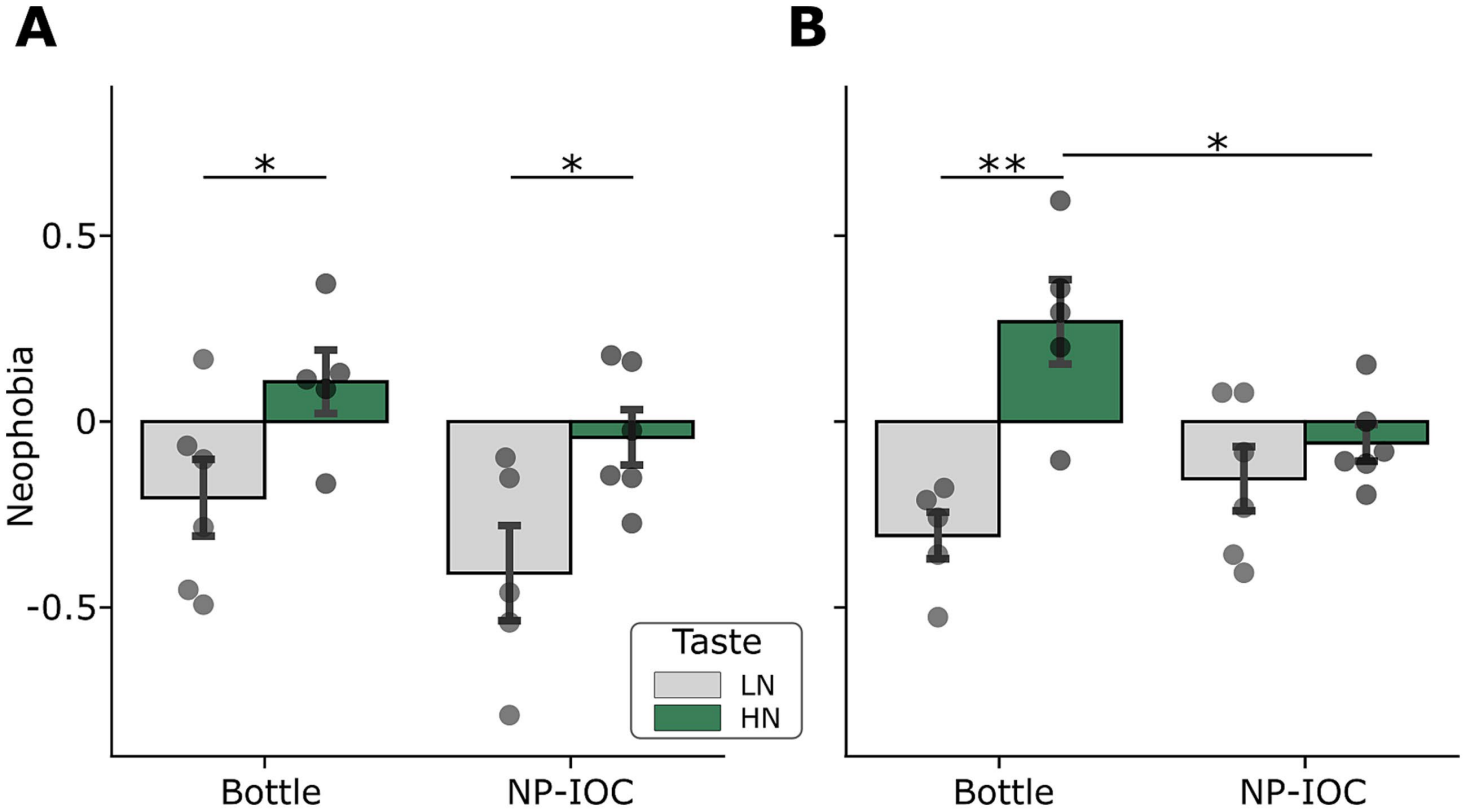
Neophobia in Bottle and NP-IOC rats toward LN and HN solutions, shown separately for males **(A)** and females **(B)**. Positive values indicate strong neophobia, while negative values indicate low neophobia or even preference, both compared to water consumption in the day prior to the neophobia experiment. Data are represented as mean ± SEM. *p < 0.05, **p < 0.01.

To further decompose the three-way interaction, we conducted follow-up analyses separately for each MOA and sex, using *post hoc* comparisons with correction for multiple testing. Under bottle administration, both males and females exhibited the expected neophobic pattern, reflected in a significantly higher neophobic reaction for the HN taste compared to the LN taste (Figures 3A,B; females: *t*(8) = −4.42, *p* = 0.002; males: *t*(9) = −2.26, *p* = 0.04). In contrast, LN consumption was associated with little or no reduction relative to water, consistent with a weak neophobic response. Under NP-IOC administration, however, neophobic responses diverged between sexes. Male rats retained the same pattern observed under bottle administration, showing a greater reduction relative to water baseline for the HN taste compared to the LN taste (Figure 3A; *t*(9) = −2.57, *p* = 0.02). In contrast, in females, the group-level difference in NN between LN and HN-exposed rats was no longer observed under NP-IOC conditions (Figure 3B; *t*(10) = −0.96, *p* = 0.35), suggesting that intake of the HN taste was no longer selectively reduced relative to water baseline. This effect was further supported by direct comparison of NN toward the HN taste across MOAs in females, which revealed a significantly weaker NN under NP-IOC compared to bottle administration (Figure 3B; *t*(9) = −2.8, *p* = 0.02). Together, these results suggest that the impact of MOA on taste neophobia is sex-dependent: while male neophobic responses remain stable across administration methods, NP-IOC selectively alters the expression of neophobia toward HN taste in females.

### 3.3 The impact of NP-IOC on CTA learning

**Figure 4:**
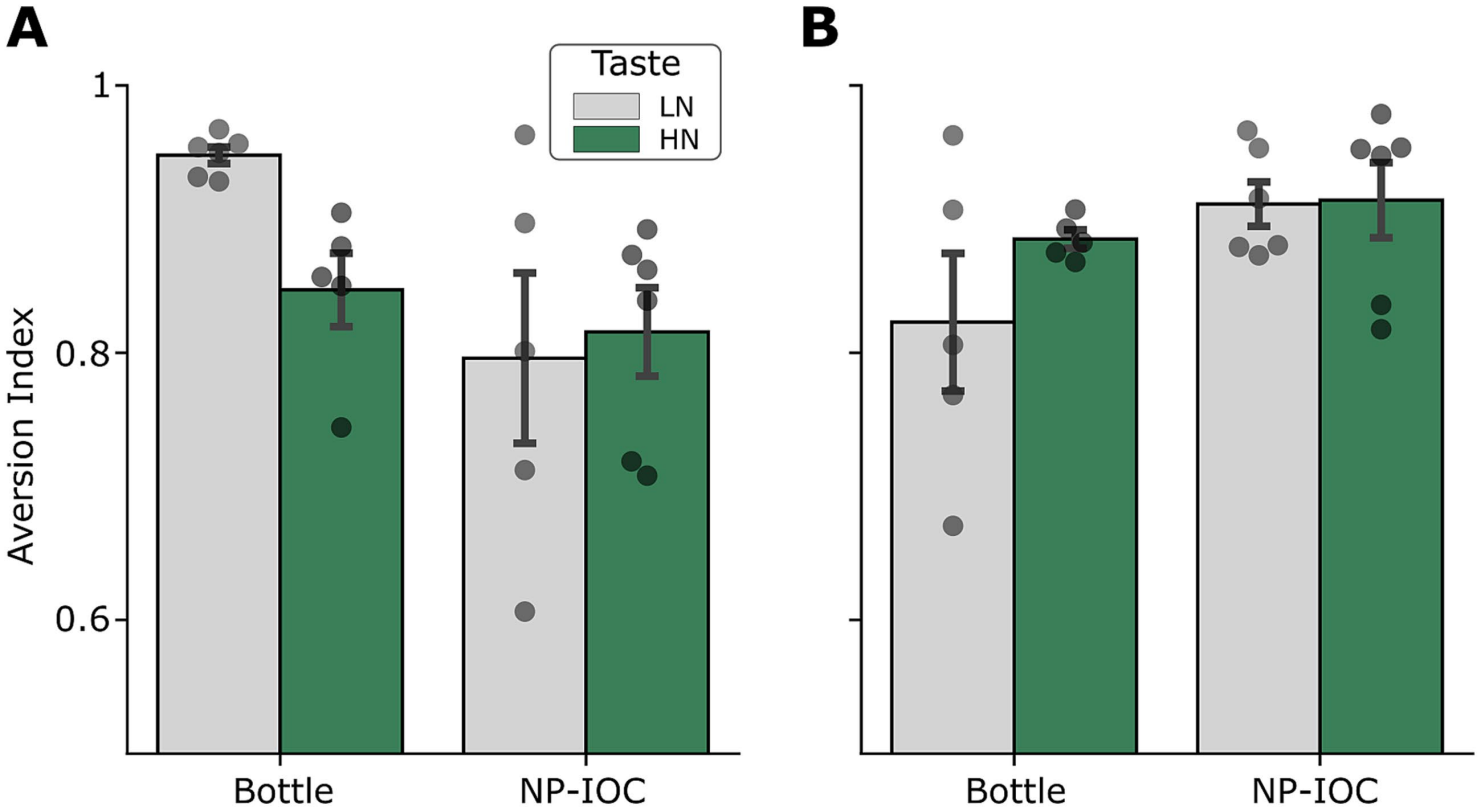
Aversion index in Bottle and NP-IOC rats toward LN and HN solutions, shown separately for males **(A)** and females **(B)**. Higher values indicate stronger aversion, reflecting greater reductions in consumption following conditioning. Data are represented as mean +/− SEM.

Next, we examined whether the NP-IOC also affects CTA learning. The strength of CTA depends on the LiCl dose, with higher doses producing stronger aversion. Here we chose to use a standard high dose that induces a robust reduction in CS consumption, consistent with previous learning studies (Arieli et al., 2020; Ramos et al., 2022; Rosenberg et al., 2016). CTA strength was quantified using the AI (Equation 2), a normalized value reflecting the reduction in taste consumption following the taste-malaise association. A significant interaction between Sex and MOA was observed (Figure 4; Three-way ANOVA Sex × Taste × MOA: F_Sex × MOA_ (1,36) = 10.26, *p* = 0.002), indicating that the effect of administration method on CTA expression differed between males and females. However, no significant main effects of Sex, Taste, or MOA were observed [F_sex_ (1,36) = 2.02, F_MOA_(1,36) = 0.49, F_Taste_(1,36) = 0.04, all *p* > 0.1], nor any significant higher-order interactions involving Taste. These results suggest that although the overall strength of aversive learning was comparable across tastes and sexes, sex-dependent differences in how CTA expression is modulated by the MOA may not be fully captured by the average AI measure alone, prompting us to further examine the relationship between pre and post-consumption across groups.

**Figure 5:**
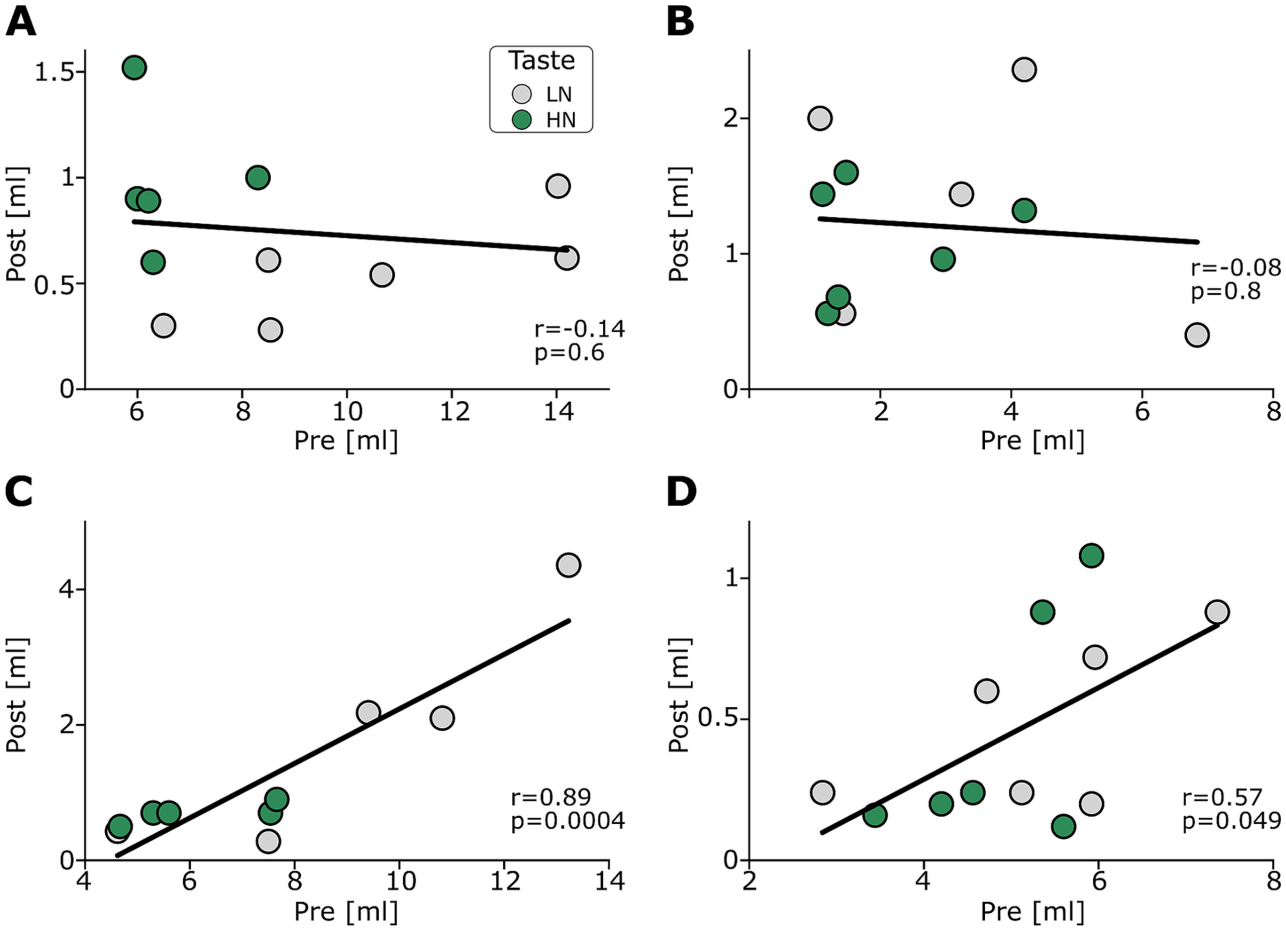
Correlation between pre- and post-CTA consumption of LN and HN tastes. Panels **(A,B)** show consumption patterns of males (**A**: bottle; **B**: NP-IOC), and panels **(C,D)** show those of females (**C**: bottle; **D**: NP-IOC).

We assessed this relationship directly using linear regression within each Sex × MOA group. As shown in Figure 5, the effect of MOA on the relation between Pre and Post-CTA consumption was different between the sexes. Similar to previous reports in male rats (Domjan and Wilson, 1972), our male rats showed a strong CTA effect irrespective of the initial pre-CTA consumption, a fact that resulted in the absence of a significant correlation under either MOA (Figures 5A,B; Pearson’s r: Bottle: *r* = −0.14, *p* = 0.6; NP-IOC: *r* = −0.08, *p* = 0.8). In contrast, females showed a strong positive correlation under bottle consumption, which was weaker but not abolished under NP-IOC (Figures 5C,D; Pearson’s *r*: Bottle: *r* = 0.89, *p* < 0.001; NP-IOC: *r* = 0.57, *p* = 0.049). Overall, these analyses demonstrate that while average CTA learning was acquired across groups, the pre-to-post consumption relationship was sex-specific: males exhibited a floor-effect-like pattern, whereas females showed a positive relation, such that higher pre-CTA intake predicted higher post-CTA consumption.

## 4 Discussion

The study of fluid consumption in animals is widely used to address diverse questions, including thirst and homeostatic regulation, reward and addiction, genetic influences on drinking behavior, and mechanisms of learning and memory. By deciding whether or how much to drink certain fluids, animals can express their internal understanding of the world and their expectations. However, the way these fluids are presented to the animals can significantly influence their consummatory decisions, consequently shaping how we interpret their behavior. In this study, we investigated the impact of the less-ethological NP-IOC method of fluid delivery on neophobic reaction to novel taste solutions and the learning of a taste-malaise association using the CTA paradigm in male and female rats. Our findings indicate that this MOA alters the neophobic response to novel tastes in a sex-specific manner. However, the fundamental process of CTA learning remains intact.

Before assessing the effect of the NP-IOC on consumption behavior, it is necessary to define how this behavior is measured, particularly in the context of neophobia. Neophobia can be defined in two ways, both of which are regularly used in research. The first approach measures neophobia as a reduction in the consumption of a novel taste relative to prior water intake, typically measured 24 h earlier (Carroll et al., 1975; Nachman and Ashe, 1974). This method is especially common when the novel taste (e.g., saccharin) is known to elicit caution or hesitation in animals. The second approach focuses on attenuation of neophobia (AON), the gradual increase in consumption of a novel taste across repeated exposures, in the absence of negative consequences (Domjan, 1976; Menchén-Márquez et al., 2026). Importantly, this method does not require initial consumption to be lower than that of water; rather, it captures the safe learning process by which animals come to accept the novel taste over time (Lin et al., 2012; Shinohara and Yasoshima, 2019). Here, we used the former definition of neophobia, which is more commonly used as it requires only a single novel taste exposure.

An additional consideration concerns the caloric differences between the taste stimuli used in this study. Sucrose and saccharin differ not only in their neophobic potential but also in energetic value, raising the possibility that caloric content may partially contribute to the observed consumption patterns. Importantly, caloric value is likely an integral component of taste-guided ingestive behavior, contributing to innate preferences and low neophobic responses toward the sweet tastes of natural sugars, as sensory properties become associated with post-ingestive consequences with experience (Sclafani, 2004). However, several lines of evidence suggest that caloric content alone cannot fully account for the neophobia effects observed here. Neophobic responses emerge rapidly during brief initial exposures to a novel taste and primarily reflect early orosensory evaluation rather than post-ingestive metabolic feedback (Gutierrez et al., 2020; Lin et al., 2012; Monk et al., 2014). In contrast, predictive associations between taste and caloric outcome that influence intake regulation typically require repeated experience across multiple sessions to develop (Swithers and Davidson, 2008). Consistent with this view, although sucrose and saccharin differ in caloric value, the sex and MOA-dependent modulation of NN observed in the present study is unlikely to be explained by energetic demand alone, suggesting that sensory and experiential factors play a central role in shaping the initial neophobic response.

When we tested the impact of MOAs on neophobia, we found that NP-IOC significantly reduced neophobia in female rats. The control groups consuming the tastes via bottles showed, as expected, stronger neophobic responses to HN compared to LN solution in both sexes (Figure 3, bottle columns). While this difference persisted in the males’ NP-IOC group, it disappeared in females. Specifically, female NP-IOC rats that consumed HN showed weak neophobia compared to bottle-fed counterparts, similar to LN consumption (Figure 3B), suggesting that NP-IOC alters the normal neophobic reaction in female rats. One potential explanation for the observed difference in neophobic responses is that intra-oral delivery via NP-IOC bypasses key sensory components of natural ingestion. Unlike bottle drinking, NP-IOC lacks olfactory input and active licking behavior, both of which play a role in flavor perception and taste-guided decisions. Taste and olfaction are tightly integrated (Malik et al., 2019), and olfactory cues have been shown to significantly influence feeding behavior in rodents (Coppola and Slotnick, 2018; Glendinning et al., 2020). Furthermore, licking is not only a motor component of ingestion, but its sensory feedback (the sensory derivative of the licking) may also serve as an active sensing signal for taste processing (Neese et al., 2022). The absence of these sensory cues in NP-IOC may reduce the perceived saliency of the HN taste, thereby weakening taste neophobia in females. Interestingly, this effect was not observed in males, whose neophobic responses continued to differentiate between LN and HN tastes under both MOAs, suggesting a sex-dependent influence of NP-IOC.

These suggestions, however, do not clarify why the MOA effect is only found in female rats. A plausible explanation is a difference in taste sensitivity between males and females. Specifically, although the difference was not statistically significant, males consumed more of the novel tastes than females via the NP-IOC (males: 7.35 mL; females: 5.32 mL), potentially resulting in longer exposure to the taste solutions. This extended exposure may have facilitated more accurate identification and processing of the taste stimuli. Indeed, repeated exposure to specific tastes has been shown to enhance taste sensitivity; for example, a taste recall training program significantly improved taste discrimination in humans following repeated exposure to various taste qualities (Otsubo et al., 2022). Such findings suggest that prolonged access to taste stimuli could similarly enhance taste perception in rodents. Supporting this interpretation, previous studies have reported that female rats are less sensitive than males to low concentrations of sucrose and NaCl, and display different preferences for sweeteners such as glucose and saccharin (Curtis et al., 2004; Flynn et al., 1993). These behavioral differences are further linked to hormonal influences and sex-specific neural responses within gustatory pathways (Martin and Sollars, 2017). Taken together, these findings support the notion that sex differences in taste sensitivity and the degree of exposure to taste stimuli may underlie the divergent neophobic behaviors observed in our study.

Exposure to a novel taste was followed by a CTA protocol to test the impact of an MOA on the formation of taste-malaise associative learning. Our results show that all experimental groups ultimately exhibited comparable levels of aversion (Figure 4), regardless of initial sex-based neophobic differences. This outcome is likely related to the high LiCl dose; this dose was selected to be consistent with widely used CTA studies linking taste learning to neural mechanisms, doses which are known to induce robust aversion, often resulting in minimal post-conditioning consumption (Nachman and Ashe, 1973). Specifically, our results in males showed a strong reduction in post-CTA consumption in both MOAs (Figures 5A,B). This reduction was independent of pre-CTA consumption; most male rats consumed only 1–2 mL of the CS taste regardless of the volume consumed pre-CTA. These results align with previous experiments showing that at this LiCl dosage, male rats develop a strong aversion response requiring only a small amount of pre-CTA taste consumption (Domjan and Wilson, 1972; Smith and Morris, 1963). In contrast, female groups exhibited a correlation between pre and post-CTA consumption in both MOA groups (Figures 5C,D). That is, while the males refrained from drinking almost any of the CS taste after CTA, females followed their initial pre-CTA drinking tendency, consuming more of the CS taste if they had consumed more during the pre-CTA session. This observed behavior in both female MOA groups has several implications. First, the consistency of these results across two different experimental conditions (the MOAs) strengthens the validity of the correlation finding. Second, it supports the use of the NP-IOC in CTA studies, as it does not appear to differ from bottle-based behaviors. Finally, it suggests a distinct behavioral difference between males and females regarding CTA learning, at least at the high LiCl dosage used here. Our findings regarding sex-specific consumption strategies align with previous reports of sexual dimorphism in taste-related behaviors, including differences in neophobia, CTA acquisition, and extinction (Angulo and Arévalo-Romero, 2021; Chambers et al., 1981; Greiner et al., 2024). These data provide further support for the hypothesis that male and female rats engage in distinct neural and behavioral strategies during taste learning (Dalla and Shors, 2009). Future studies incorporating additional tastants, varying LiCl dosages, and extended exposures (e.g., AON) will help clarify the underlying mechanisms of these sex differences, especially under NP-IOC conditions.

In summary, our findings highlight the importance of methodological considerations in studies of taste learning. The NP-IOC method provides precise control over stimulus delivery time while maintaining voluntary choice, establishing it as a valuable tool for investigating taste behaviors. Furthermore, the sex-specific effects observed here underscore the urgent need to treat sex as a key biological variable and to increase the inclusion of females in behavioral research, a practice that has historically been understudied. Together, these results not only validate NP-IOC as a reliable behavioral assay but also call for future studies to directly investigate the neural and behavioral mechanisms underlying sex differences in taste learning.

## Data Availability

The raw data supporting the conclusions of this article will be made available by the authors, without undue reservation.
